# Microscopy of the Teeth

**Published:** 1868-01

**Authors:** S. P. Cutler


### MICROSCOPY OF THE TEETH—CONTINUED.

BY S. P. CUTLER, M. D., A. E. G., D. D. S.

Professor of Chemistry and Microscopy in the Ohio Dental College, Cincinnati, formerly Professor of Chemistry and Na'ural Sci-nces in the Botanic Medical College, Memphis, Tenn.

I find I am always treading on somebody's sore toes, consequently I must try and blaze out some new and unfrequented route to travel in, which I hope I have found at last; at least, so far as it has come to my knowledge. I mean the microscopic crystals that make their appearance in specimens prepared from fresh pulps.

When a section is taken from a fresh pulp and pressed between two slips of glass, slightly numerous fat globules make their appearance, very small, regular and round. On pressing the glasses closer together, these fat discs are more or less broken up, and running together form larger ones of various sizes, all of which are round, some of them several times larger than the blood discs, possessing strong optical properties.

These globules occupy more or less the whole specimen. What proportion of fat and other proximate principles these globules contain has not yet been determined. There is yet much to learn concerning the chemical equivalents of the pulp.

After a time there begins to flow out from the specimen, minute streams or currents drawn out from the fluid elements by capillary attraction of the surfaces of the glasses and by pressure combined. The size of these streams are governed by the relative spaces between the glasses.

These currents flow out in all directions, crossing each other in various directions; sometimes continuing in straight lines, sometimes in curves, forming a complete reticulated structure, beautiful in the extreme; presenting after a few days a cell like and baccated structure, something like fibrine when treated with twigs while coagulating.

In some instances at the terminus of some of these streams perfect links or beads in contact, form themselves into coils of unusual beauty and interest as microscopic specimens. In some instances clusters of these small bodies collect at the ends of short and rather broad streams resembling cauliflower; some groups larger others smaller. These small bodies are perfectly regular, generally slightly oval and flattened and diafenous.

In these specimens portions of the system of nerves of the pulp and the openings through the pulp membrane may be seen. No crystals are to be seen in these currents or secondary formations.

In the above formations, have we not a typal of histology in these apparent cell structures, from the vital forces of the proximate principles that are not yet extinct at the time of these formations.

In specimens properly prepared, after a certain time numerous crystals form more or less extensively throughout the specimen; sometimes they form only around the border of the specimen, shooting out in a radiate or stellate form; sometimes shooting out from certain points from a dark centre. In such specimens fewer crystals are observed throughout.

In some instances scattering ones may be seen just outside of the specimen, fusiform in shape in some instances, in others only one end is pointed, the other presenting a broken appearance.

The crystals that form in the specimen itself, are generally in the form of double bundles branching out from near the centre; at each end branching out in fine, circular forms resembling almost exactly the crystals of stearin and margarin, and those of the glyko cholate of soda of the bile, presenting the same general phenomenon.

These crystals in some specimens are scattered extensively throughout, always in isolated groups; in some cases they may be seen in stellated groups, surrounding a dark ground centre, sending off very short and fine needles. These crystals are exceedingly beautiful under the microscope; they are no doubt formed from the stearine and margarine of the pulp, and the amount of crystals in each specimen are in proportion to the amount of these elements. These crystals are formed during the desication or drying of the specimen, which takes place in the course of a day or two, varying in different specimens.

I do not recollect of having seen a description of them by any one.

These would have been described before the ossified pulp, only the subject was not then sufficiently matured to venture an opinion.

In my next article I will give my mode of preparing the specimen for this purpose.

[TO BE CONTINUED.]

